# Cholesterol and Copper Affect Learning and Memory in the Rabbit

**DOI:** 10.1155/2013/518780

**Published:** 2013-08-29

**Authors:** Bernard G. Schreurs

**Affiliations:** ^1^Blanchette Rockefeller Neurosciences Institute, West Virginia University, Morgantown, WV 26505, USA; ^2^Department of Physiology and Pharmacology, West Virginia University, P.O. Box 9302, Morgantown, WV 26506, USA

## Abstract

A rabbit model of Alzheimer's disease based on feeding a cholesterol diet for eight weeks shows sixteen hallmarks of the disease including beta amyloid accumulation and learning and memory changes. Although we have shown that feeding 2% cholesterol and adding copper to the drinking water can retard learning, other studies have shown that feeding dietary cholesterol before learning can improve acquisition and feeding cholesterol after learning can degrade long-term memory. We explore the development of this model, the issues surrounding the role of copper, and the particular contributions of the late D. Larry Sparks.

## 1. Introduction

In 2001, we were looking for nontransgenic animal models of Alzheimer's disease (AD) in which we could study the effects of potential treatments on AD deficits in learning and memory. A review of the literature revealed very few options other than aged animals that would take many months or even years to reach a point at which they could be studied [1–4]. One exception was a cholesterol-fed rabbit model of AD that Sparks and colleagues showed had several hallmarks of Alzheimer's pathology, particularly beta amyloid accumulation, that developed in as a little as 8 weeks of being fed a 2% cholesterol diet [5–8]. Surprisingly, given the well-characterized rabbit eyeblink conditioning preparation first published by Gormezano and colleagues in the 1960s [9–13], there were no studies in the literature examining learning and memory in these cholesterol-fed rabbits. We contacted Larry Sparks to ask why no one had published learning and memory studies with this model and the answer was as clear and emphatic as only Larry Sparks could make it: he had tried to convince researchers for years to do the experiments but no one seemed to be interested. 

 One possible reason for this apparent lack of interest in studying learning and memory in a rabbit model of AD was the fact that standard rabbit eyeblink conditioning experiments in which a tone preceded and overlapped with a puff of air to the eye was mediated in large part by the cerebellum [14, 15], and the cerebellum is the last and least affected brain structure in patients with AD [16]. However, this mediation of learning by the cerebellum is only true for the most basic of classical conditioning paradigms known as delay conditioning in which the stimuli overlap [17]. If there is a substantial trace between the two stimuli and the tone and air puff do not overlap, there is good evidence that the hippocampus and prefrontal cortex are engaged and become critical to successful learning and memory [18–30]. The hippocampus and cortex are among the areas that are the first and most profoundly affected structures in patients with AD [16, 31]. 

## 2. The Effects of Cholesterol on Learning

 In collaboration with Sparks, we sought to assess the effects of a cholesterol diet on trace conditioning of the rabbit nictitating membrane response [32]. The results of these first experiments with the nictitating membrane response (NMR) were to start us on a ten-year odyssey that still continues to challenge us and has been deeply affected by the untimely death of our colleague Larry Sparks.

 In order to study NMR conditioning in cholesterol-fed rabbits, we instituted a standard set of procedures that began with rabbits being fed 2% cholesterol or standard Purina rabbit chow (0% cholesterol) for eight weeks and then presented the rabbits with pairings of a brief tone (100 ms, 82 dB, 1 kHz) as the conditioned stimulus (CS) followed by an eyeblink-eliciting air puff (100 ms, 4 psi) or periorbital electrical pulse (100 ms, 2.0 mA, 60 Hz) as the unconditioned stimulus (US). In some experiments, half of the rabbits received explicitly unpaired presentations of the CS and US to assess nonassociative contributors to responding [9, 13, 33–36]. Importantly, the interval between the CS and US for paired rabbits was more than 500 ms creating a significant trace which previous studies by a number of groups have shown made classical conditioning dependent on the hippocampus [18–20, 37–40] and prefrontal cortex [23, 25, 27, 29, 41–45] in addition to the cerebellum. In each of our trace conditioning experiments, acquisition of a conditioned response was a function of the trace interval and usually took many days of training to reach asymptote, and this asymptote tended to be lower than that seen using delay conditioning [17]. Importantly, subsequent delay conditioning and sensory thresholds were always the same for cholesterol-fed rabbits and normal chow controls [32, 46]. Cholesterol-fed unpaired control subjects showed low levels of responding that were consistent with previous observations in rabbits fed normal chow [9, 33, 47]. In all of these experiments, the cholesterol diet continued throughout the course of the behavioral manipulations. 

 With the previously well-documented accumulation of intracellular beta amyloid induced by feeding 2% cholesterol for 8 weeks reported by Sparks and his colleagues [5, 6, 8], the first experiments we conducted were surprising because of the expectation that we would see a beta amyloid-induced deficit in learning when, in fact, we saw a facilitation of NMR conditioning [32]. This is a finding we have seen in many of our subsequent experiments [48–50]. The facilitated conditioning was indexed by higher levels of responding to the CS [32, 48] and heightened responsivity to the US measured after conditioning, and known as a conditioning-specific reflex modification [32, 48–50]. When the levels of beta amyloid accumulation in our cholesterol-fed rabbits were examined by the Sparks laboratory, the immunoreactivity was relatively light although significantly higher than in the rabbits fed normal chow. At that point, we discussed the results with Sparks and it became clear that there was an as yet untold part of the story involving the drinking water given to rabbits.

## 3. The Contents of Tap Water

 The effects of drinking water on beta amyloid accumulation have been discussed at length in a number of articles by Sparks and colleagues and will only be summarized here [51–53]. The original finding that the contents of tap water might be important to the level of beta amyloid immunoreactivity began with the observation by Sparks that after moving his laboratory from Kentucky to Arizona, cholesterol-fed rabbits showed significantly less intense beta amyloid immunoreactivity. Upon investigation, and ruling out other potential causes, it came to light that the rabbits in the Arizona facility were being given distilled water to drink and, when they were returned to tap water, the beta amyloid immunoreactivity became more intense [51]. An analysis of trace metals in Morgantown tap water by an independent laboratory showed virtually no detectable levels of copper [32]. In contrast, rabbits that had been switched to and maintained on tap water in Arizona showed high levels of beta amyloid immunoreactivity and high levels of copper in the tap water [32, 51]. Subsequent manipulation of copper in distilled drinking water showed that the level of beta amyloid immunoreactivity was, indeed, a function of copper in the drinking water [46, 51–54]. In his own inimitable style, Sparks initially broached the entire subject of the role of drinking water in beta amyloid accumulation when he began a conversation about our initial light beta amyloid staining results with “And now for the rest of the story…”.

## 4. The Effects of Copper on Learning

**Figure 1:**
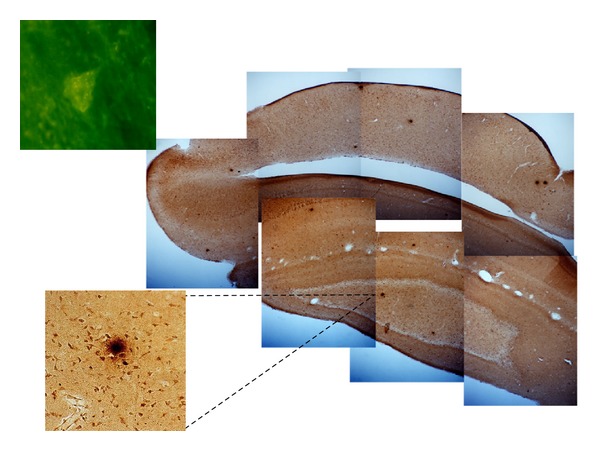
Photo montage of temporal lobe and hippocampus for a cholesterol-fed rabbit given distilled water supplemented with 0.12 parts per million copper as copper sulfate (right). Note the numerous dark spots that appear to be plaque-like structures that are shown in detail at bottom left. The top left image shows a thioflavin-S stained neuron from a cholesterol-fed rabbit given distilled water supplemented with 0.12 parts per million copper.

**Figure 2:**
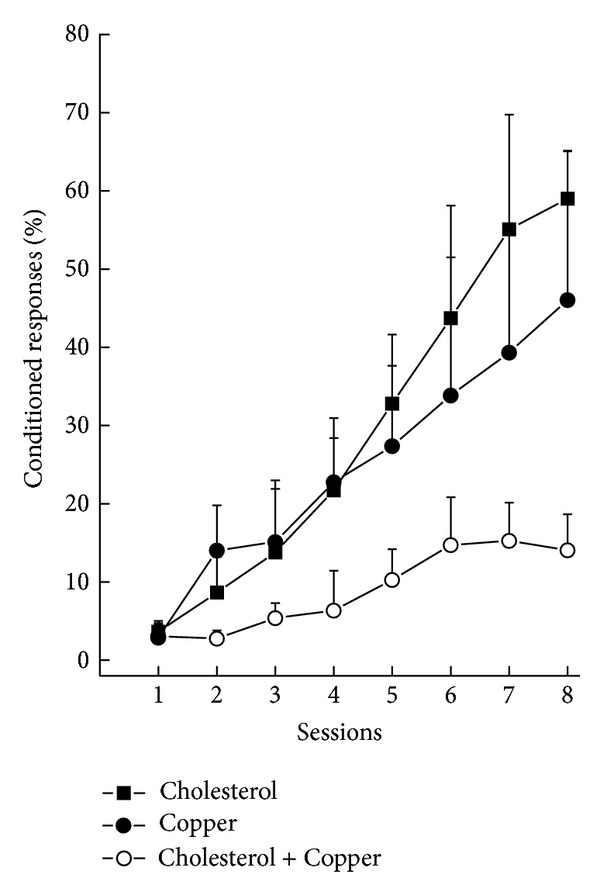
Mean percent (±SEM) conditioned responses to a tone conditioned stimulus as a function of eight days of pairings (sessions) of the tone and air puff to the eye for rabbits fed 2% cholesterol in their rabbit chow (Cholesterol), fed normal chow and given 0.12 parts per million copper as copper sulfate in their distilled drinking water (Copper), or fed 2% cholesterol and given 0.12 parts per million copper in their distilled drinking water (Cholesterol + Copper). The data show lower levels of trace conditioning of the nictitating membrane response in rabbits fed cholesterol and given copper in their drinking water. The data are modified from Sparks and Schreurs [46].

We next conducted a seminal experiment [46] in which we added 0.12 parts per million (ppm) copper as copper sulfate to distilled drinking water and found that the levels of beta amyloid in cholesterol-fed rabbits had increased over previous levels to the point of generating extracellular plaques and, importantly, these rabbits showed a deficit in trace conditioning relative to controls. The photo montage in Figure 1 shows evidence of extracellular plaque-like structures as well as dense intracellular immunoreactivity to the beta amyloid antibody 10D5 shown in detail at the bottom left of Figure 1. Figure 1 also shows a thioflavin-S stained neuron in detail (top left) in a rabbit fed cholesterol and given copper in its drinking water.  Figure 2 shows that the level of trace conditioning acquired by rabbits given cholesterol and copper was significantly lower than rabbits fed cholesterol and given distilled water and rabbits fed normal chow and given copper in their drinking water. Although there is a suggestion that the cholesterol-fed animals drinking distilled water might have had higher terminal levels of responding than rabbits fed normal chow, the differences were not significant. As noted above, all rabbits were able to acquire a simple delay conditioning task in which the tone and air puff overlapped and all showed very similar auditory thresholds indicating that the cholesterol and copper did not affect sensory processing or simple delay conditioning [46]. The essential aspects of these initial findings of increased beta amyloid and lower levels of trace eyeblink conditioning were subsequently replicated by another rabbit conditioning laboratory [55, 56]. The beta amyloid accumulation resulting from rabbits being fed cholesterol has also been independently confirmed by the Ghribi group who have gone on to study some of the underlying molecular mechanisms [57–60].

**Figure 3:**
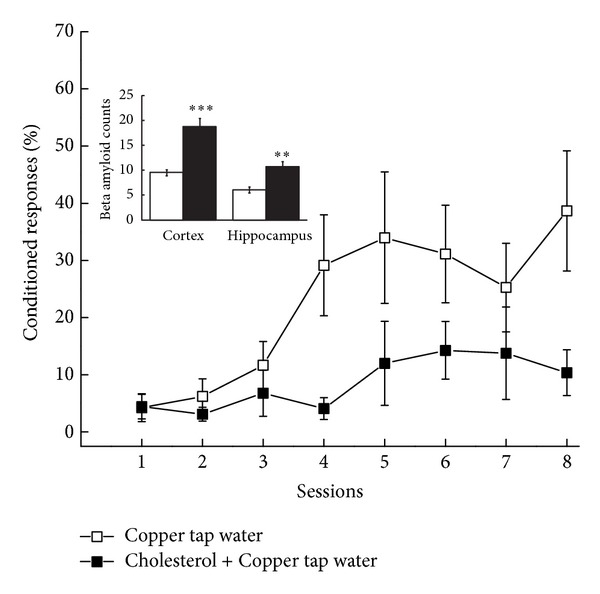
Mean percent (±SEM) conditioned responses to a tone conditioned stimulus as a function of eight days of pairings (Sessions) of the tone and air puff to the eye for rabbits fed normal rabbit chow and given 0.12 parts per million copper as copper sulfate in their tap water (Copper Tap Water) or fed 2% cholesterol and given 0.12 parts per million copper in their tap water (Cholesterol + Copper Tap Water). The inset shows the mean (±SEM) number of counts of beta amyloid immunoreactive cells in the cortex and hippocampus. The data show lower levels of trace conditioning of the nictitating membrane response and number of beta amyloid positive cells in rabbits fed cholesterol and given copper in their tap water.

To further explore the role of copper and tap water, we conducted a simple experiment in which we fed rabbits 2% cholesterol and provided them with Morgantown tap water which had been supplemented with 0.12 ppm copper. These rabbits were compared to rabbits that were fed normal chow and given tap water supplemented with 0.12 ppm copper. The data in Figure 3 show both the level of responding during trace conditioning and the number of beta amyloid immune-positive neurons in the cortex and hippocampus of the two groups. Figure 3 shows and statistical analysis confirmed that the level of trace conditioning acquired by rabbits given cholesterol and copper in tap water was significantly lower than rabbits fed chow and given tap water with copper (*P* < .005). Once again all rabbits were able to acquire delay conditioning and showed identical auditory thresholds indicating that the cholesterol and copper did not affect sensory processing or simple response acquisition. The inset of Figure 3 shows that the number of beta amyloid immune-positive cells was significantly higher in the cortex and hippocampus of rabbits fed cholesterol and given copper in tap water than those fed chow. These data help confirm the original findings shown in Figure 2 and suggest that there may be more to the effects of water than first thought. For example, a subsequent analysis of the tap water supplemented with 0.12 ppm copper stored in standard carboys for four-five weeks yielded a large number of components similar to our original analysis of the tap water in Morgantown [32] but, surprisingly, the level of copper was only 0.085 ppm. The level of copper in tap water supplemented with 0.12 ppm copper and stored for only two weeks was 0.104 ppm. Clearly, storage in polypropylene carboys caused significant changes in copper concentration as a function of time and, as a result, we instituted weekly preparation of fresh copper-supplemented water that was administered to rabbits in glass bottles. 

## 5. Cholesterol, Copper, and Beta Amyloid

At this point, we began a series of parametric experiments in which we manipulated the concentration [48] and duration [50] of cholesterol and routinely included copper in the distilled drinking water. We continued to see the facilitating effects of cholesterol on NMR conditioning [48, 50] but, surprisingly, we did not see the debilitating effects on NMR conditioning with the addition of copper to the cholesterol. Importantly, although we continued to see higher levels of beta amyloid immunoreactivity with copper added to the drinking water of cholesterol-fed rabbits compared to those on distilled water, there was no evidence of extracellular beta amyloid plaques. This was even true when we doubled the copper concentration in the drinking water to 0.24 ppm although, in that case, the cortical levels of beta amyloid immunoreactivity were significantly higher in chow-fed rabbits given 0.24 ppm copper compared to those given distilled water suggesting that copper by itself was having an effect on beta amyloid accumulation [61]. 

During this second phase of behavioral experiments, a heightened sensitivity by veterinary staff to the hepatotoxic effects of the cholesterol diet [53, 62–64] meant that animals were being given supplementary feeding or withdrawn from studies earlier and more often than had occurred in our original studies. It is possible that the beta amyloid load was not as severe and the consequent extracellular plaque formations were no longer being detected because of this earlier withdrawal from the studies. In a separate development, the Sparks laboratory began to notice a decrease in the intensity of beta amyloid staining with the 10D5 antibody and the problem became worse with succeeding batches of antibody. Nevertheless, in an unpublished study, we continue to find beta amyloid immunoreactivity with a commercial human beta amyloid enzyme linked-immunosorbent assay and immunofluorescent labeling based on the 6E10 beta amyloid antibody. Moreover, other groups have also reported significant elevations in beta amyloid as a result of feeding rabbits cholesterol [55, 58, 65, 66].

## 6. Imaging the Effects of Cholesterol and Copper

**Figure 4:**
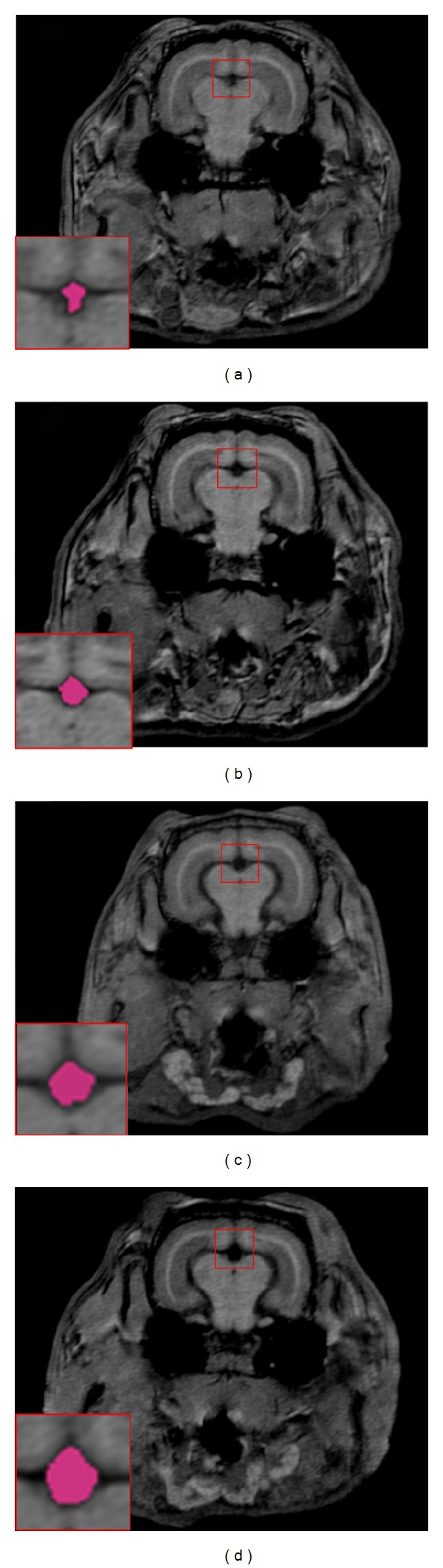
Structural MRI scan of a rabbit that received normal chow and distilled drinking water (a), normal chow and 0.12 ppm copper added to the distilled drinking water (b), 2% cholesterol and distilled drinking water (c), or 2% cholesterol and copper added to the distilled drinking water (d). The inset shows the area around the third ventricle in each rabbit and the red shading illustrates the significantly larger area of the third ventricle for cholesterol-fed rabbits ((c) and (d)) compared to the chow-fed control rabbits ((a) and (b)). The data show significant increases in the area of the third ventricle as a function of being fed cholesterol. Data are modified from [72].

**Figure 5:**
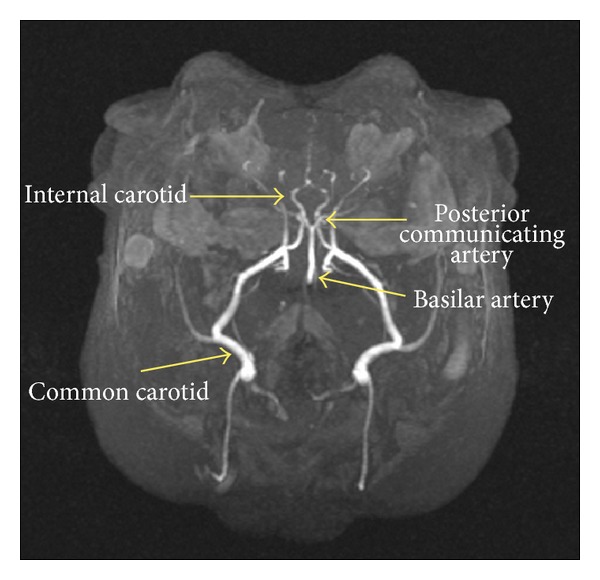
Blood vessels in the rabbit brain that were visualized by time-of-flight magnetic resonance angiography during our MRI studies and analysis of vessel diameters focused on the left and right common carotid arteries, the basilar artery, the left and right internal carotids arteries, and the left and right posterior communicating arteries.

**Figure 6:**
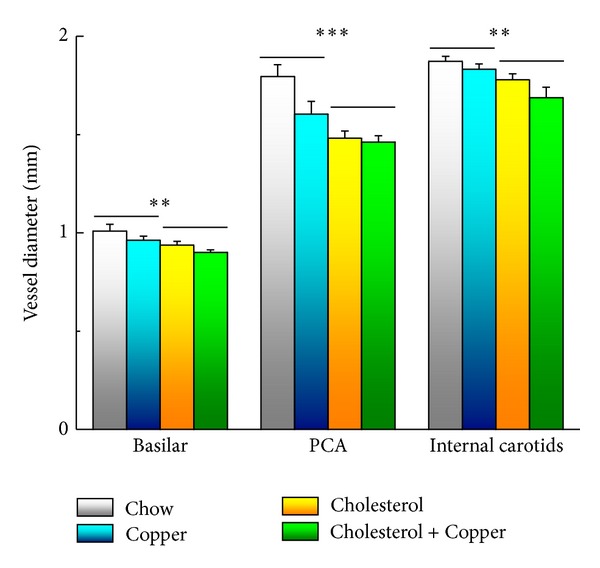
Mean (±SEM) vessel diameter of the left and right internal carotid arteries, basilar artery, and left and right posterior communicating arteries (PCA) for rabbits fed normal chow and given distilled drinking water (Chow), 2% cholesterol in their rabbit chow and given distilled drinking water (Cholesterol), fed normal chow and given 0.12 parts per million copper as copper sulfate in their distilled drinking water (Copper), or fed 2% cholesterol and given 0.12 parts per million copper in their distilled drinking water (Cholesterol + Copper). Data are modified from [72].

 At the conclusion of many of our behavioral experiments, we began structural MRI imaging of the rabbits' brains to explore the effects of cholesterol and copper on rabbit ventricular volume [61, 67] and cerebrovascular diameter [67]—indices that have been noted to change in patients with AD [68–71]. The four panels of Figure 4 show structural MRI scans of rabbits that received normal chow and distilled water (a), normal chow and 0.12 ppm copper added to the distilled water (b), 2% cholesterol and distilled water (c), and 2% cholesterol and copper (d), with insets that show the area of the third ventricle. The data in Figure 4 illustrate clearly that a cholesterol diet significantly increased the area of the third ventricle and consequently, when the entire rabbit brain was analyzed, the volume of the third ventricle was found to be higher for the cholesterol-fed rabbits than the normal chow-fed controls [72]. This was true regardless of whether the rabbits were given copper in the drinking water or whether the concentration of that copper was 0.12 ppm [72] or 0.24 ppm [61]. In all of these experiments, the levels of beta amyloid immunoreactivity to the 10D5 antibody was always higher in cholesterol-fed rabbits than normal chow controls, and the addition of copper tended to increase the intensity of that immunoreactivity even further although this copper-induced increase was not always significantly higher [48–50, 61].  Figure 5 shows the blood vessels in a rabbit brain that were visualized by time-of-flight angiography during our MRI studies and they include the common carotid arteries, the basilar artery, the internal carotids, and the posterior communicating arteries [67].  Figure 6 shows that the basilar, internal carotid, and posterior communicating arteries were all narrowed by a 2% cholesterol diet compared to normal chow controls and that the addition of 0.12 ppm copper to the drinking water did not significantly increase the narrowing [72]. There were no significant differences in diameter of the common carotid arteries.

## 7. The Effects of Cholesterol on Other Forms of Learning

 As important as the behavioral effects of cholesterol were with the rabbit NMR, there were two broader questions. First, would the effects of cholesterol on acquisition of the rabbit NMR hold true for other forms of learning, and second, what were the effects of cholesterol on memory? Although the majority of the research into learning in rabbits is based on changes in skeletal responses, particularly the closure of the upper eyelid and the sweep of the nictitating membrane [35], a significant body of research has examined an autonomic response—deceleration of heart rate [73–80]. The adult rabbit typically shows an unconditioned increase in heart rate (HR) to electrical stimulation and an unconditioned decrease in HR to tones. The unconditioned HR acceleration to electrodermal stimulation is an acute response to a stressful stimulus and has been used as a measure of the animal's “defense reaction” [80]. The unconditioned deceleration in HR to a tone is an orienting response that can be habituated by tone-alone presentations. Heart rate classical conditioning occurs when rabbits that receive pairings of tone and shock show a conditioned deceleration in HR to the tone relative to rabbits that show little or no change in HR when they receive explicitly unpaired tone and shock presentations [73, 81, 82]. This type of autonomic conditioning usually occurs within a relatively few pairings that consist of tones that are separated from shock by a second or more [83]. Using a trace conditioning paradigm, we found that cholesterol facilitated rabbit HR conditioning and that the unconditioned HR response to shock was also modified by conditioning [49]. The significant facilitation of HR conditioning suggests that the effects of cholesterol on learning were not specific to one form of conditioning involving a skeletal response but to an autonomic response as well. Importantly, a major anatomical locus for rabbit HR conditioning is the amygdala [75, 84–86] and we found that, in addition to the hippocampus and cortex, the level of beta amyloid staining in the amygdala was higher in cholesterol-fed rabbits than in controls [49].

 Our findings of cholesterol-induced facilitated learning are consistent with experiments in a number of other animal models that have reported that modifying dietary cholesterol can improve learning [87]. For example, increasing cholesterol in mutant mice in which hippocampally dependent spatial learning is normally impaired improves performance in the Morris water maze [88, 89]. Feeding cholesterol to young, normal rats also improves performance in the Morris water maze [90, 91]. Feeding cholesterol to rats that are either deficient in cholesterol or have cholesterol synthesis blocked reverses problems with learning in the water maze and acquisition of eyeblink conditioning [92–95]. These animal data are also consistent with some human literature showing that higher cognitive functioning is correlated with high cholesterol [96, 97] and that cholesterol may protect against cognitive decline especially in the elderly [97–100]. 

## 8. The Effects of Cholesterol on Memory

 The majority of research with humans suggests strongly that cholesterol is detrimental to memory. A significant number of studies show that elevated serum cholesterol is a risk factor for mild cognitive impairment [101–105] and dementia [106, 107] and that cholesterol levels are correlated with measures of intelligence [103, 108–111] except in the very elderly [99, 112]. Low HDL cholesterol has been correlated with deficits and declines in memory in midlife [113]. A study of cholesterol synthesis showed the level of the cholesterol precursors lanosterol and lathosterol are correlated with low memory performance as subjects age [114]. It is to this second question—the effects of cholesterol on memory—that we next turned our attention.

 In an experiment by Darwish and colleagues, we trained rabbits to asymptotic levels of NMR tracing conditioning and then instituted an 8-week diet of 2% cholesterol or normal chow before assessing the memory of trace conditioning by presenting the tone alone over a period of days during extinction [115]. Rabbits fed normal chow showed response levels of about 60% at the beginning of extinction which was very consistent with a previous assessment of long-term memory for trace conditioning of the rabbit NMR after 8 weeks [116]. In contrast, cholesterol-fed rabbits showed significantly lower response levels of only 30%—a level that was not significantly higher than cholesterol-fed unpaired control rabbits [115]. We were able to replicate this finding with different concentrations of cholesterol that all showed lower levels of responding during extinction than a normal chow control group [117]. Importantly, neither of these experiments involved the addition of copper to the distilled drinking water suggesting that cholesterol by itself can degrade a previously acquired memory. 

## 9. The Effects of Cholesterol and Copper on Both Learning and Memory

 In a recent study, we combined our behavioral procedures and cholesterol feeding regimens into a single paradigm. We used discrimination reversal conditioning to assess the effects of a cholesterol diet and copper in the water on the memory of a previously acquired association and the ability of the rabbits to acquire a new and opposite association. In brief, rabbits were trained to discriminate between two tones of different frequency (1 kHz and 8 kHz), then placed on an 8-week cholesterol diet with or without 0.12 ppm copper in their drinking water, then tested for their memory of the original discrimination, and subsequently trained to reverse that discrimination [118–122]. The data showed that cholesterol and distilled water degraded the ability of rabbits to remember the original discrimination but facilitated their ability to learn the reversal of that discrimination. Interestingly, the addition of copper to the water of cholesterol-fed rabbits had the opposite effect on both phenomena—the rabbits were able to recall the original discrimination but were less able to learn the reversal of that discrimination. Importantly, a rabbit's ability to successfully reverse a discrimination is dependent on an intact, functioning hippocampus which allows the rabbit to inhibit responding to a previously paired stimulus [118, 120]. We found that cholesterol-fed rabbits were better able to inhibit responding than cholesterol-fed rabbits given copper and have shown elsewhere that the membrane excitability of hippocampal neurons is increased by cholesterol feeding that is decreased by the addition of copper to the drinking water [123]. Membrane excitability has been shown to increase as a function of learning [124–128]. Taken together, these data show that cholesterol by itself and cholesterol supplemented by copper have opposite effects on behavior as well as on one of the underlying neural mechanisms associated with learning and memory.

## 10. Effect of Cholesterol and Copper on Beta Amyloid Accumulation and Learning and Memory in This Model

 It is clear that feeding rabbits cholesterol increases the level of beta amyloid in the brain at the same time that it has significant systemic effects particularly in the liver and vasculature [87, 129–133]. Our research shows that against a backdrop of increased beta amyloid immunoreactivity in the cortex and hippocampus, there are replicable effects of feeding cholesterol on learning and memory. Given the essential role of the cortex and hippocampus in the acquisition and recall of trace conditioning in both eyelid and heart conditioning [26, 39, 55, 134–138], it is tempting to draw causal inferences from the accumulation of beta amyloid in those structures and the observed changes in learning and memory particularly given our findings of cholesterol-induced changes in the membrane properties of hippocampal neurons [123]. In fact, this recapitulates the inferences the field continues to make concerning beta amyloid and Alzheimer's disease. However, the recent very public failures of clinical trials designed to mitigate the effects of beta amyloid have given some researchers grounds to revisit other potential mechanisms that may play a part in the development of Alzheimer's disease including the role of vascular factors, inflammation, oxidative stress, and the role of trace metals including copper, to name a few. For example, there is strong evidence that human cognitive impairment is correlated with the extent of cholesterol-induced atherosclerosis both in peripheral arterial disease [139] and in carotid atherosclerosis [140]. There is also a growing awareness of the effects of inflammation on cognitive decline [141–144]. Similarly, there are a significant number of peripheral effects of cholesterol in the rabbit that may have effects on learning and memory including atherosclerosis [145–147], inflammation [5, 133, 148, 149], oxidative stress [150–152], and copper [153–155]. With the compromise of the rabbit's blood brain barrier that occurs with cholesterol feeding [5, 58, 156–158], these peripheral effects may very well also become central effects. Finally, beta amyloid is present in the brain from birth to death and, in normal concentrations, is critical for cell function, synaptic plasticity, and memory [159]. In other words, there are a number of complex effects of cholesterol, copper, and beta amyloid and it is probably a combination or interaction of several of these effects that can best explain their influence on learning and memory.

## 11. Summary

 From our first published rabbit NMR study [32], the majority of experiments have shown that cholesterol facilitates learning and the addition of copper, which increases the level of beta amyloid immunoreactivity, reverses this facilitation, and, in at least two cases, makes it significantly worse than controls [46]. More recently, we have found that cholesterol degrades long-term memory both of simple acquisition as well as discrimination learning, and, in the latter case, copper returns responding to control levels. On the other hand, as noted above, the addition of copper to cholesterol tends to exacerbate the level of beta amyloid immunoreactivity but only slightly increases other indices of pathology including increases in ventricular volume and cerebrovascular diameter beyond those induced by cholesterol alone [61, 67]. Taken together, these results have raised a number of important questions including the nature of the effects of cholesterol on learning and memory, the potential mechanisms of these effects, and the role copper may have in modifying the effects of cholesterol and in elevating beta amyloid.

## References

[1] Bi X, Head E, Cotman CW, Lynch G (2003). Spatial patterns of mammalian brain aging: distribution of cathepsin D-immunoreactive cell bodies and dystrophic dendrites in aging dogs resembles that in Alzheimer’s disease. *Journal of Comparative Neurology*.

[2] Cotman CW, Head E, Muggenburg BA, Zicker S, Milgram NW (2002). Brain aging in the canine: a diet enriched in antioxidants reduces cognitive dysfunction. *Neurobiology of Aging*.

[3] Johnstone EM, Chaney MO, Norris FH, Pascual R, Little SP (1991). Conservation of the sequence of the Alzheimer’s disease amyloid peptide in dog, polar bear and five other mammals by cross-species polymerase chain reaction analysis. *Molecular Brain Research*.

[4] McDonald MP, Overmier JB (1997). Present imperfect: a critical review of animal models of the mnemonic impairments in Alzheimer’s disease. *Neuroscience and Biobehavioral Reviews*.

[5] Sparks DL, Kuo Y-M, Roher A, Martin T, Lukas RJ (2000). Alterations of Alzheimer’s disease in the cholesterol-fed rabbit, including vascular inflammation. Preliminary observations. *Annals of the New York Academy of Sciences*.

[6] Sparks DL (1997). Dietary cholesterol induces Alzheimer-like *β*-amyloid immunoreactivity in rabbit brain. *Nutrition, Metabolism and Cardiovascular Diseases*.

[7] Sparks DL, Liu H, Gross DR, Scheff SW (1995). Increased density of cortical apolipoprolein; immunoreactive neurons in rabbit brain after dietary administration of cholesterol. *Neuroscience Letters*.

[8] Sparks DL, Scheff SW, Hunsaker JC, Liu H, Landers T, Gross DR (1994). Induction of Alzheimer-like *β*-amyloid immunoreactivity in the brains of rabbits with dietary cholesterol. *Experimental Neurology*.

[9] Gormezano I, Schneiderman N, Deaux E, Fuentes I (1962). Nictitating membrane: classical conditioning and extinction in the albino rabbit. *Science*.

[10] Schneiderman N, Fuentes I, Gormezano I (1962). Acquisition and extinction of the classically conditioned eyelid response in the albino rabbit. *Science*.

[11] Deaux EB, Gormezano I (1963). Eyeball retraction: classical conditioning and extinction in the albino rabbit. *Science*.

[12] Schneiderman N, Gormezano I (1964). Conditioning of the nictitating membrane of the rabbit as a function of CS-US interval. *Journal of Comparative and Physiological Psychology*.

[13] Gormezano I, Sidowski JB (1966). Classical conditioning. *Experimental Methods and Instrumentation in Psychology*.

[14] McCormick DA, Thompson RF (1984). Cerebellum: essential involvement in the classically conditioned eyelid response. *Science*.

[15] Yeo CH, Hardiman MJ, Glickstein M (1984). Discrete lesions of the cerebellar cortex abolish the classically conditioned nictitating membrane response of the rabbit. *Behavioural Brain Research*.

[16] Thal DR, Rüb U, Orantes M, Braak H (2002). Phases of A*β*-deposition in the human brain and its relevance for the development of AD. *Neurology*.

[17] Schneiderman N (1966). Interstimulus interval function of the nictitating membrane response of the rabbit under delay versus trace conditioning. *Journal of Comparative and Physiological Psychology*.

[18] Port RL, Mikhail AA, Patterson MM (1985). Differential effects of hippocampectomy on classically conditioned rabbit nictitating membrane response related to interstimulus interval. *Behavioral Neuroscience*.

[19] Soloman PR, Vander Schaaf ER, Thompson RF, Weisz DJ (1986). Hippocampus and trace conditioning of the rabbit’s classically conditioned nictitating membrane response. *Behavioral Neuroscience*.

[20] Moyer JR, Deyo RA, Disterhoft JF (1990). Hippocampectomy disrupts trace eye-blink conditioning in rabbits. *Behavioral Neuroscience*.

[21] Steinmetz JE, Lavond DG, Ivkovich D, Logan CG, Thompson RF (1992). Disruption of classical eyelid conditioning after cerebellar lesions: damage to a memory trace system or a simple performance deficit?. *Journal of Neuroscience*.

[22] Kim JJ, Clark RE, Thompson RF (1995). Hippocampectomy impairs the memory of recently, but not remotely, acquired trace eyeblink conditioned responses. *Behavioral Neuroscience*.

[23] Kronforst-Collins MA, Disterhoft JF (1998). Lesions of the caudal area of rabbit medial prefrontal cortex impair trace eyeblink conditioning. *Neurobiology of Learning and Memory*.

[24] Gruart A, Morcuende S, Martínez S, Delgado-García JM (2000). Involvement of cerebral cortical structures in the classical conditioning of eyelid responses in rabbits. *Neuroscience*.

[25] Weible AP, McEchron MD, Disterhoft JF (2000). Cortical involvement in acquisition and extinction of trace eyeblink conditioning. *Behavioral Neuroscience*.

[26] Ryou J-W, Cho S-Y, Kim H-T (2001). Lesions of the entorhinal cortex impair acquisition of hippocampal-dependent trace conditioning. *Neurobiology of Learning and Memory*.

[27] McLaughlin J, Skaggs H, Churchwell J, Powell DA (2002). Medial prefrontal cortex and Pavlovian conditioning: trace versus delay conditioning. *Behavioral Neuroscience*.

[28] Wikgren J, Ruusuvirta T, Korhonen T (2003). Activity in the rabbit somatosensory cortex reflects the active procedural memory trace of a classically conditioned eyeblink response. *Neuroscience Letters*.

[29] Powell DA, Churchwell J, Burriss L (2005). Medial prefrontal lesions and Pavlovian eyeblink and heart rate conditioning: effects of partial reinforcement on delay and trace conditioning in rabbits (*Oryctolagus cuniculus*). *Behavioral Neuroscience*.

[30] Woodruff-Pak DS, Disterhoft JF (2008). Where is the trace in trace conditioning?. *Trends in Neurosciences*.

[31] Braak H, Braak E (1995). Staging of Alzheimer’s disease-related neurofibrillary changes. *Neurobiology of Aging*.

[32] Schreurs BG, Smith-Bell CA, Lochhead J, Sparks DL (2003). Cholesterol modifies classical conditioning of the rabbit (*Oryctolagus cuniculus*) nictitating membrane response. *Behavioral Neuroscience*.

[33] Gormezano I, Kehoe EJ, Estes WK (1975). Classical conditioning: some methodological-conceptual issues. *Handbook of Learning and Cognitive Processes*.

[34] Gormezano I, Kehoe EJ, Harzem P (1981). Classical conditioning and the law of contiguity. *Predictability, Correlation, and Contiguity*.

[35] Gormezano I, Kehoe EJ, Marshall BS, Sprague JM (1983). Twenty years of classical conditioning research with the rabbit. *Progress in Psychobiology and Physiological Psychology*.

[36] Gormezano I, Alkon DL (1984). The study of associative learning with CS-CR paradigms. *Primary Neural Substrates of Learning and Behavioral Change*.

[37] Seager MA, Asaka Y, Berry SD (1999). Scopolamine disruption of behavioral and hippocampal responses in appetitive trace classical conditioning. *Behavioural Brain Research*.

[38] McEchron MD, Disterhoft JF (1999). Hippocampal encoding of non-spatial trace conditioning. *Hippocampus*.

[39] McEchron MD, Tseng W, Disterhoft JF (2000). Neurotoxic lesions of the dorsal hippocampus disrupt auditory-cued trace heart rate (fear) conditioning in rabbits. *Hippocampus*.

[40] Thompson RF (2005). In search of memory traces. *Annual Review of Psychology*.

[41] Simon B, Knuckley B, Churchwell J, Powell DA (2005). Post-training lesions of the medial prefrontal cortex interfere with subsequent performance of trace eyeblink conditioning. *Journal of Neuroscience*.

[42] Oswald B, Knuckley B, Mahan K, Sanders C, Powell DA (2006). Prefrontal control of trace versus delay eyeblink conditioning: role of the unconditioned stimulus in rabbits (*Oryctolagus cuniculus*). *Behavioral Neuroscience*.

[43] Kalmbach BE, Ohyama T, Kreider JC, Riusech F, Mauk MD (2009). Interactions between prefrontal cortex and cerebellum revealed by trace eyelid conditioning. *Learning & Memory*.

[44] Oswald BB, Maddox SA, Tisdale N, Powell DA (2010). Encoding and retrieval are differentially processed by the anterior cingulate and prelimbic cortices: a study based on trace eyeblink conditioning in the rabbit. *Neurobiology of Learning and Memory*.

[45] Suter EE, Weiss C, Disterhoft JF (2013). Perirhinal and postrhinal, but not lateral entorhinal, cortices are essential for acquisition of trace eyeblink conditioning. *Learning & Memory*.

[46] Sparks DL, Schreurs BG (2003). Trace amounts of copper in water induce *β*-amyloid plaques and learning deficits in a rabbit model of Alzheimer’s disease. *Proceedings of the National Academy of Sciences of the United States of America*.

[47] Smith MC, Coleman SR, Gormezano I (1969). Classical conditioning of the rabbit’s nictitating membrane response at backward, simultaneous, and forward CS-US intervals. *Journal of Comparative and Physiological Psychology*.

[48] Schreurs BG, Smith-Bell CA, Darwish DS, Stankovic G, Sparks DL (2007). High dietary cholesterol facilitates classical conditioning of the rabbit’s nictitating membrane response. *Nutritional Neuroscience*.

[49] Schreurs BG, Smith-Bell CA, Darwish DS (2007). Cholesterol enhances classical conditioning of the rabbit heart rate response. *Behavioural Brain Research*.

[50] Schreurs BG, Smith-Bell CA, Darwish DS, Stankovic G, Sparks DL (2007). Classical conditioning of the rabbit’s nictitating membrane response is a function of the duration of dietary cholesterol. *Nutritional Neuroscience*.

[51] Sparks DL, Lochhead J, Horstman D, Wagoner T, Martin T (2002). Water quality has a pronounced effect on cholesterol-induced accumulation of Alzheimer amyloid *β* (A*β*) in rabbit brain. *Journal of Alzheimer’s Disease*.

[52] Sparks DL, Friedland R, Petanceska S (2006). Trace copper levels in the drinking water, but not zinc or aluminum influence CNS Alzheimer-like pathology. *Journal of Nutrition, Health and Aging*.

[53] Sparks DL, Martin T, Stankovic G, Wagoner T, Van Andel R (2007). Influence of water quality on cholesterol induced systemic pathology. *Journal of Nutrition, Health and Aging*.

[54] Sparks DL (2004). Cholesterol, copper, and accumulation of thioflavine S-reactive Alzheimer’s-like amyloid *β* in rabbit brain. *Journal of Molecular Neuroscience*.

[55] Woodruff-Pak DS, Agelan A, Valle LD (2007). A rabbit model of Alzheimer’s disease: valid at neuropathological, cognitive, and therapeutic levels. *Journal of Alzheimer’s Disease*.

[56] Coico R, Woodruff-Pak DS (2008). Immunotherapy for Alzheimer’s disease: harnessing our knowledge of T cell biology using a cholesterol-fed rabbit model. *Journal of Alzheimer’s Disease*.

[57] Ghribi O, Larsen B, Schrag M, Herman MM (2006). High cholesterol content in neurons increases BACE, *β*-amyloid, and phosphorylated tau levels in rabbit hippocampus. *Experimental Neurology*.

[58] Ghribi O, Golovko MY, Larsen B, Schrag M, Murphy EJ (2006). Deposition of iron and *β*-amyloid plaques is associated with cortical cellular damage in rabbits fed with long-term cholesterol-enriched diets. *Journal of Neurochemistry*.

[59] Sharma S, Prasanthi R.P. J, Schommer E, Feist G, Ghribi O (2008). Hypercholesterolemia-induced A*β* accumulation in rabbit brain is associated with alteration in IGF-1 signaling. *Neurobiology of Disease*.

[60] Jaya Prasanthi RP, Schommer E, Thomasson S, Thompson A, Feist G, Ghribi O (2008). Regulation of *β*-amyloid levels in the brain of cholesterol-fed rabbit, a model system for sporadic Alzheimer’s disease. *Mechanisms of Ageing and Development*.

[61] Deci S, Lemieux SK, Smith-Bell CA, Sparks DL, Schreurs BG (2012). Cholesterol increases ventricular volume in a rabbit model of alzheimer’s disease. *Journal of Alzheimer’s Disease*.

[62] Ho KJ, Pang LC, Liu LB (1974). Cholesterol accumulation in various rabbits’ tissues with variations in serum levels and duration of exposure. *Experimental and Molecular Pathology*.

[63] Song S-H, Min B-I, Lee J-H, Cho KS (2000). Chronological effects of atherogenic diets on the aorta, liver and spleen of rabbits. *Journal of Korean Medical Science*.

[64] De Wolf ID, Fielmich-Bouman XM, Lankhorst A (2003). Cholesterol and copper in the liver of rabbit inbred strains with differences in dietary cholesterol response. *Journal of Nutritional Biochemistry*.

[65] Beach TG (2008). Physiologic origins of age-related *β*-amyloid deposition. *Neurodegenerative Diseases*.

[66] Ronald JA, Chen Y, Bernas L (2009). Clinical field-strength MRI of amyloid plaques induced by low-level cholesterol feeding in rabbits. *Brain*.

[67] Lemieux SK, Smith-Bell CA, Wells JR (2010). Neurovascular changes measured by time-of-flight MR angiography in cholesterol-fed rabbits with cortical amyloid *β*-peptide accumulation. *Journal of Magnetic Resonance Imaging*.

[68] Leung KK, Barlett JW, Manning EN, Ourselin S, Fox NC (2013). Cerbral atrophy in mild cognitive impairment and Alzheimer disease. *Neurology*.

[69] Nestor SM, Rupsingh R, Borrie M (2008). Ventricular enlargement as a possible measure of Alzheimer’s disease progression validated using the Alzheimer’s disease neuroimaging initiative database. *Brain*.

[70] Bell RD (2012). The imbalance of vascular molecules in Alzheimer's disease. *Journal of Alzheimer's Disease*.

[71] Sagare AP, Bell RD, Zlokovic BV (2012). Neurovascular dysfunction and faulty amyloid *β*-peptide clearance in Alzheimer disease. *Cold Spring Harbor Perspectives in Medicine*.

[72] Schreurs BG, Smith-Bell CA, Lemieux SK (2013). Dietary cholesterol increases ventricular volume and narrows cerebrovascular diameter in a rabbit model of Alzheimer's Disease. *Neuroscience*.

[73] Schneiderman N, Reynierse JH (1970). Determinants of heart rate classical conditioning. *Current Issues in Animal Learning: A Colloquium*.

[74] Powell DA, Kazis E (1976). Blood pressure and heart rate changes accompanying classical eyeblink conditioning in the rabbit (*Oryctolagus cuniculus*). *Psychophysiology*.

[75] Applegate CD, Frysinger RC, Kapp BS, Gallagher M (1982). Multiple unit activity recorded from amygdala central nucleus during Pavlovian heart rate conditioning in rabbit. *Brain Research*.

[76] McEchron MD, McCabe PM, Green EJ, Llabre MM, Schneiderman N (1992). Air puff versus shock unconditioned stimuli in rabbit heart rate conditioning. *Physiology and Behavior*.

[77] Supple WF, Kapp BS (1993). The anterior cerebellar vermis: essential involvement in classically conditioned bradycardia in the rabbit. *Journal of Neuroscience*.

[78] Sebastiani L, Salamone D, Silvestri P, Simoni A, Ghelarducci B (1994). Development of fear-related heart rate responses in neonatal rabbits. *Journal of the Autonomic Nervous System*.

[79] Ghelarducci B, Salamone D, Simoni A, Sebastiani L (1996). Effects of early cerebellar removal on the classically conditioned bradycardia of adult rabbits. *Experimental Brain Research*.

[80] Schreurs BG, Crum JM, Wang D, Smith-Bell CA (2005). Conditioning-specific reflex modification of rabbit (*Oryctolagus cuniculus*) heart rate. *Behavioral Neuroscience*.

[81] Schneiderman N, Black AH, Prokasy WF (1972). Response system divergencies in aversive classical conditioning. *Classical Conditioning II: Current Research and Theory*.

[82] Kazis E, Milligan WL, Powell DA (1973). Autonomic somatic relationships: blockade of heart rate and corneo retinal potential responses. *Journal of Comparative and Physiological Psychology*.

[83] McEchron MD, Tseng W, Disterhoft JF (2003). Single neurons in CA1 hippocampus encode trace interval duration during trace heart rate (fear) conditioning in rabbit. *Journal of Neuroscience*.

[84] McCabe PM, Schneiderman N, Jarrell TW, Gormezano I, Wasserman EA (1992). Central pathways involved in classical differential conditioning of heart rate responses in rabbits. *Learning and Memory: The Behavioral and Biological Substrates*.

[85] Powell DA, Tebbutt D, Chachich M, Murphy V, McLaughlin J, Buchanan SL (1997). Amygdala-prefrontal interactions and conditioned bradycardia in the rabbit. *Behavioral Neuroscience*.

[86] Kim JJ, Jung MW (2006). Neural circuits and mechanisms involved in Pavlovian fear conditioning: a critical review. *Neuroscience and Biobehavioral Reviews*.

[87] Schreurs BG (2010). The effects of cholesterol on learning and memory. *Neuroscience and Biobehavioral Reviews*.

[88] Miller S, Wehner JM (1994). Cholesterol treatment facilitates spatial learning performance in DBA/2Ibg mice. *Pharmacology Biochemistry and Behavior*.

[89] Upchurch M, Wehner JM (1988). DBA/2Ibg mice are incapable of cholinergically-based learning in the Morris water task. *Pharmacology Biochemistry and Behavior*.

[90] Dufour F, Liu Q-Y, Gusev P, Alkon D, Atzori M (2006). Cholesterol-enriched diet affects spatial learning and synaptic function in hippocampal synapses. *Brain Research*.

[91] Ya BL, Liu WY, Ge F, Zhang YX, Zhu BL, Bai B (2012). Dietary cholesterol alters memory and synaptic structural plasticity in young rat brain. *Neurological Science*.

[92] Võikar V, Rauvala H, Ikonen E (2002). Cognitive deficit and development of motor impairment in a mouse model of Niemann-Pick type C disease. *Behavioural Brain Research*.

[93] Xu G, Servatius RJ, Shefer S (1998). Relationship between abnormal cholesterol synthesis and retarded learning in rats. *Metabolism: Clinical and Experimental*.

[94] Endo Y, Nishimura J-I, Kimura F (1996). Impairment of maze learning in rats following long-term glucocorticoid treatments. *Neuroscience Letters*.

[95] O’Brien WT, Xu G, Batta A (2002). Developmental sensitivity of associative learning to cholesterol synthesis inhibitors. *Behavioural Brain Research*.

[96] Elias PK, Elias MF, D’Agostino RB, Sullivan LM, Wolf PA (2005). Serum cholesterol and cognitive performance in the Framingham Heart Study. *Psychosomatic Medicine*.

[97] Panza F, D’Introno A, Colacicco AM (2006). Lipid metabolism in cognitive decline and dementia. *Brain Research Reviews*.

[98] Mielke MM, Zandi PP, Sjögren M (2005). High total cholesterol levels in late life associated with a reduced risk of dementia. *Neurology*.

[99] West R, Beeri MS, Schmeidler J (2008). Better memory functioning associated with higher total and low-density lipoprotein cholesterol levels in very elderly subjects without the apolipoprotein e4 allele. *American Journal of Geriatric Psychiatry*.

[100] van den Kommer TN, Dik MG, Comijs HC, Fassbender K, Lütjohann D, Jonker C (2009). Total cholesterol and oxysterols: early markers for cognitive decline in elderly?. *Neurobiology of Aging*.

[101] Kivipelto M, Helkala E-L, Hänninen T (2001). Midlife vascular risk factors and late-life mild cognitive impairment: a population-based study. *Neurology*.

[102] Näslund J, Haroutunian V, Mohs R (2000). Correlation between elevated levels of amyloid *β*-peptide in the brain and cognitive decline. *Journal of the American Medical Association*.

[103] Yaffe K, Barrett-Connor E, Lin F, Grady D (2002). Serum lipoprotein levels, statin use, and cognitive function in older women. *Archives of Neurology*.

[104] Foster TC (2006). Biological markers of age-related memory deficits: treatment of senescent physiology. *CNS Drugs*.

[105] Solomon A, Kåreholt I, Ngandu T (2007). Serum cholesterol changes after midlife and late-life cognition: twenty-one-year follow-up study. *Neurology*.

[106] Whitmer RA, Sidney S, Selby J, Claiborne Johnston S, Yaffe K (2005). Midlife cardiovascular risk factors and risk of dementia in late life. *Neurology*.

[107] Solomon A, Kivipelto M, Wolozin B, Zhou J, Whitmer RA (2009). Midlife serum cholesterol and increased risk of Alzheimer’s and vascular dementia three decades later. *Dementia and Geriatric Cognitive Disorders*.

[108] Reitan RM, Shipley RE (1963). The relationship of serum cholesterol changes to psychological abilities. *Journal of Gerontology*.

[109] Muldoon MF, Ryan CM, Matthews KA, Manuck SB (1997). Serum cholesterol and intellectual performance. *Psychosomatic Medicine*.

[110] Van Exel E, De Craen AJM, Gussekloo J (2002). Association between high-density lipoprotein and cognitive impairment in the oldest old. *Annals of Neurology*.

[111] Atzmon G, Gabriely I, Greiner W, Davidson D, Schechter C, Barzilai N (2002). Plasma HDL levels highly correlate with cognitive function in exceptional longevity. *Journals of Gerontology*.

[112] Solomon A, Kåreholt I, Ngandu T (2009). Serum total cholesterol, statins and cognition in non-demented elderly. *Neurobiology of Aging*.

[113] Singh-Manoux A, Gimeno D, Kivimaki M, Brunner E, Marmot MG (2008). Low HDL cholesterol is a risk factor for deficit and decline in memory in midlife the whitehall II study. *Arteriosclerosis, Thrombosis, and Vascular Biology*.

[114] Teunissen CE, De Vente J, Von Bergmann K (2003). Serum cholesterol, precursors and metabolites and cognitive performance in an aging population. *Neurobiology of Aging*.

[115] Darwish DS, Wang D, Konat GW, Schreurs BG (2010). Dietary cholesterol impairs memory and memory increases brain cholesterol and sulfatide levels. *Behavioral Neuroscience*.

[116] Schreurs BG (1998). Long-term memory and extinction of rabbit nictitating membrane trace conditioning. *Learning and Motivation*.

[117] Schreurs BG, Wang D, Smith-Bell CA, Burhans LB, Bell R, Gonzales-Joekes J (2012). Dietary cholesterol concentration and duration degrade long-term memory of classical conditioning of the rabbit's nictitating membrane response. *International Journal of Alzheimer's Disease*.

[118] Berger TW, Orr WB (1983). Hippocampectomy selectively disrupts discrimination reversal conditioning of the rabbit nictitating membrane response. *Behavioural Brain Research*.

[119] Gould TJ, Steinmetz JE (1994). Multiple-unit activity from rabbit cerebellar cortex and interpositus nucleus during classical discrimination/reversal eyelid conditioning. *Brain Research*.

[120] Miller DP, Steinmetz JE (1997). Hippocampal activity during classical discrimination—reversal eyeblink conditioning in rabbits. *Behavioral Neuroscience*.

[121] Churchill JD, Green JT, Voss SE, Manley E, Steinmetz JE, Garraghty PE (2001). Discrimination reversal conditioning of an eyeblink response is impaired by NMDA receptor blockade. *Integrative Physiological and Behavioral Science*.

[122] Nokia MS, Wikgren J (2010). Hippocampal theta activity is selectively associated with contingency detection but not discrimination in rabbit discrimination-reversal eyeblink conditioning. *Hippocampus*.

[123] Wang D, Schreurs BG (2010). Dietary cholesterol modulates the excitability of rabbit hippocampal CA1 pyramidal neurons. *Neuroscience Letters*.

[124] Moyer JR, Thompson LT, Disterhoft JF (1996). Trace eyeblink conditioning increases CA1 excitability in a transient and learning-specific manner. *Journal of Neuroscience*.

[125] Zhu L, Scelfo B, Tempia F, Sacchetti B, Strata P (2006). Membrane excitability and fear conditioning in cerebellar Purkinje cell. *Neuroscience*.

[126] Kim SJ, Linden DJ (2007). Ubiquitous plasticity and memory storage. *Neuron*.

[127] Bekisz M, Garkun Y, Wabno J, Hess G, Wrobel A, Kossut M (2010). Increased excitability of cortical neurons induced by associative learning: an ex vivo study. *European Journal of Neuroscience*.

[128] Schreurs BG, Gusev PA, Tomsic D, Alkon DL, Shi T (1998). Intracellular correlates of acquisition and long-term memory of classical conditioning in Purkinje cell dendrites in slices of rabbit cerebellar lobule HVI. *Journal of Neuroscience*.

[129] Williams KJ, Feig JE, Fisher EA (2008). Rapid regression of atherosclerosis: insights from the clinical and experimental literature. *Nature Clinical Practice Cardiovascular Medicine*.

[130] Sparks DL (2008). The early and ongoing experience with the cholesterol-fed rabbit as a model of Alzheimer’s disease: the old, the new and the pilot. *Journal of Alzheimer’s Disease*.

[131] Yanni AE (2004). The laboratory rabbit: an animal model of atherosclerosis research. *Laboratory Animals*.

[132] Moghadasian MH (2002). Experimental atherosclerosis: a historical overview. *Life Sciences*.

[133] Stokes KY, Cooper D, Tailor A, Granger DN (2002). Hypercholesterolemia promotes inflammation and microvascular dysfunction: role of nitric oxide and superoxide. *Free Radical Biology and Medicine*.

[134] Morrissey MD, Maal-Bared G, Brady S, Takehara-Nishiuchi K (2012). Functional dissociation within the entorhinal cortex for memory retrieval of an association between temporally discontiguous stimuli. *Journal of Neuroscience*.

[135] Green JT, Arenos JD (2007). Hippocampal and cerebellar single-unit activity during delay and trace eyeblink conditioning in the rat. *Neurobiology of Learning and Memory*.

[136] Chowdhury N, Quinn JJ, Fanselow MS (2005). Dorsal hippocampus involvement in trace fear conditioning with long, but not short, trace intervals in mice. *Behavioral Neuroscience*.

[137] Weible AP, Weiss C, Disterhoft JF (2003). Activity profiles of single neurons in caudal anterior cingulate cortex during trace eyeblink conditioning in the rabbit. *Journal of Neurophysiology*.

[138] Weiss C, Bouwmeester H, Power JM, Disterhoft JF (1999). Hippocampal lesions prevent trace eyeblink conditioning in the freely moving rat. *Behavioural Brain Research*.

[139] Rafnsson SB, Deary IJ, Fowkes FGR (2009). Peripheral arterial disease and cognitive function. *Vascular Medicine*.

[140] Romero JR, Beiser A, Seshadri S (2009). Carotid artery atherosclerosis, MRI indices of brain ischemia, aging, and cognitive impairment: the framingham study. *Stroke*.

[141] Perry VH (2010). Contribution of systemic inflammation to chronic neurodegeneration. *Acta Neuropathologica*.

[142] Holmes C, Cunningham C, Zotova E (2009). Systemic inflammation and disease progression in Alzheimer disease. *Neurology*.

[143] Solfrizzi V, D’Introno A, Colacicco AM (2006). Circulating biomarkers of cognitive decline and dementia. *Clinica Chimica Acta*.

[144] Yaffe K, Kanaya A, Lindquist K (2004). The metabolic syndrome, inflammation, and risk of cognitive decline. *Journal of the American Medical Association*.

[145] Riedmüller K, Metz S, Bonaterra GA (2010). Cholesterol diet and effect of long-term withdrawal on plaque development and composition in the thoracic aorta of New Zealand White rabbits. *Atherosclerosis*.

[146] de Prada TP, Pozzi AO, Coronado MT (2007). Atherogenesis takes place in cholesterol-fed rabbits when circulating concentrations of endogenous cortisol are increased and inflammation suppressed. *Atherosclerosis*.

[147] Russell JC, Proctor SD (2006). Small animal models of cardiovascular disease: tools for the study of the roles of metabolic syndrome, dyslipidemia, and atherosclerosis. *Cardiovascular Pathology*.

[148] Xue Q-S, Sparks DL, Streit WJ (2007). Microglial activation in the hippocampus of hypercholesterolemic rabbits occurs independent of increased amyloid production. *Journal of Neuroinflammation*.

[149] Fan J, Watanabe T (2003). Inflammatory reactions in the pathogenesis of atherosclerosis. *Journal of Atherosclerosis and Thrombosis*.

[150] Prasad K, McNair ED, Qureshi AM, Casper-Bell G (2012). Vitamin E slows the progression of hypercholesterolemia-induced oxidative stress in heart, liver, and kidney. *Molecular and Cellular Biochemistry*.

[151] Bolayirli IM, Aslan M, Balci H, Altug T, Hacibekiroglu M, Seven A (2007). Effects of atorvastatin therapy on hypercholesterolemic rabbits with respect to oxidative stress, nitric oxide pathway and homocysteine. *Life Sciences*.

[152] Collin B, Busseuil D, Zeller M (2007). Increased superoxide anion production is associated with early atherosclerosis and cardiovascular dysfunctions in a rabbit model. *Molecular and Cellular Biochemistry*.

[153] Rashtchizadeh N, Ettehad S, DiSilvestro RA, Mahdavi R (2008). Antiatherogenic effects of zinc are associated with copper in iron-overloaded hypercholesterolemic rabbits. *Nutrition Research*.

[154] Rajendran R, Ren M, Ning P, Tan Kwong Huat B, Halliwell B, Watt F (2007). Promotion of atherogenesis by copper or iron-Which is more likely?. *Biochemical and Biophysical Research Communications*.

[155] Lamb DJ, Tickner ML, Hourani SMO, Ferns GAA (2005). Dietary copper supplements modulate aortic superoxide dismutase, nitric oxide and atherosclerosis. *International Journal of Experimental Pathology*.

[156] Jiang X, Guo M, Su J (2012). Simvastatin blocks blood-brain barrier disruptions induced by elevated cholesterol both in vivo and in vitro. *International Journal of Alzheimer’s Disease*.

[157] Chen X, Gawryluk JW, Wagener JF, Ghribi O, Geiger JD (2008). Caffeine blocks disruption of blood brain barrier in a rabbit model of Alzheimer’s disease. *Journal of Neuroinflammation*.

[158] Ong W-Y, Tan B, Pan N (2004). Increased iron staining in the cerebral cortex of cholesterol fed rabbits. *Mechanisms of Ageing and Development*.

[159] Puzzo D, Privitera L, Fa’ M (2011). Endogenous amyloid-*β* is necessary for hippocampal synaptic plasticity and memory. *Annals of Neurology*.

